# Divergent temporal patterns in adolescent behavioural risk factors across sex, age, and region in Indonesia: a national repeated cross-sectional study, 2007–2023

**DOI:** 10.1080/16549716.2026.2752484

**Published:** 2026-10-29

**Authors:** Aprezo Pardodi Maba, Elizabeth Edwards, Asaduzzaman Khan

**Affiliations:** a School of Health and Rehabilitation Sciences, The University of Queensland, Brisbane, Australia; b Faculty of Education and Teacher Training, Universitas Ma’arif Lampung, Metro, Indonesia; c School of Education, The University of Queensland, Brisbane, Australia

**Keywords:** Alcohol use, fruit and vegetable intake, Indonesian adolescent, physical activity, tobacco use

## Abstract

**Background:**

National surveillance evidence describing long-term changes in adolescent behavioural risk factors in Indonesia remains limited.

**Objective:**

To examine changes in tobacco use, alcohol use, insufficient physical activity, and insufficient fruit and vegetable intake between 2007 and 2023.

**Methods:**

We analysed nationally representative Indonesian surveys from 2007, 2013, 2018, and 2023, comprising 653,700 adolescents aged 10–18 years. Survey-weighted estimates described population prevalence, and Poisson regression with robust standard errors estimated temporal differences adjusted for sex, age, and region.

**Results:**

Tobacco use increased to 2018 before returning to baseline by 2023. Alcohol use prevalence was broadly comparable in 2007 and 2018, but substantially lower in 2023. Insufficient physical activity was markedly lower in 2013 than in other years; the 2018 and 2023 estimates were closer to 2007. Insufficient fruit and vegetable intake declined to 2018, before rising above baseline by 2023. Tobacco and alcohol use were higher among males and older adolescents. Insufficient physical activity was higher among females and younger adolescents, whereas insufficient fruit and vegetable intake was higher among females and older adolescents. Papua had the highest combined tobacco prevalence from 2013 onward, largely reflecting higher chewing tobacco use; Kepulauan Nusa Tenggara had the highest alcohol prevalence. Temporal changes varied by age and geographic region across all outcomes.

**Conclusion:**

Adolescent behavioural risk factors showed divergent changes, with persistent demographic and regional differences. Findings support targeted tobacco and alcohol prevention for high-risk groups and regions, alongside population-based strategies through schools, families, and communities to improve physical activity and diet.

## Paper Context

- **Main findings**: Over 16 years, Indonesia achieved limited overall improvement in adolescent behavioural health, with declining alcohol use but tobacco use returning close to baseline, no sustained improvement in physical activity, and worsening insufficient fruit and vegetable intake.
- **Added knowledge**: This study shows that improvement in one behavioural domain did not necessarily coincide with improvement in others. Prevalence differed by sex, age, and geographic region, while temporal trends varied by age and region across all four outcomes.
- **Global health impact for policy and action**: Adolescent health strategies should adopt a two-track approach, targeting tobacco and alcohol prevention among males, older adolescents, and adolescents in higher-prevalence regions, while strengthening school-, family-, and community-based strategies to improve physical activity and diet.

## Background

Adolescence represents a critical developmental period during which behavioural risk factors such as tobacco use, alcohol consumption, insufficient physical activity, and unhealthy dietary behaviours become established. Tobacco and alcohol use during adolescence are associated with increased risk of substance dependence and continued engagement in health-risk behaviours later in life [1]. Whereas insufficient physical activity and poor dietary behaviours contribute to the development of obesity, cardiovascular disease, and type 2 diabetes [2]. These behaviours form part of the broader ‘*Big-6*’ adolescent lifestyle risk behaviours, which also include sedentary behaviour and inadequate sleep, and are recognised as major contributors to long-term non-communicable disease risk and adolescent health burden [3]. Beyond their implications for physical health, these behaviours have also been linked to poorer mental wellbeing, reduced academic functioning, and substantial long-term economic burden [4–6]. Behavioural risks do not emerge uniformly across adolescent populations; rather, their prevalence varies across demographic and geographic characteristics [7,8]. Adolescent behavioural risk factors may also follow different temporal patterns, with some remaining widespread while others become increasingly concentrated within particular populations across time [9,10]. As a result, behavioural risks may become unevenly distributed across adolescent populations, increasing the burden within some sociodemographic groups. Understanding how these patterns change over time is therefore important for adolescent health surveillance and prevention planning.

In Indonesia, nationally representative datasets, including the Basic Health Research (RISKESDAS), Indonesian Health Survey (SKI), the Global School-based Student Health Survey, and the Global Youth Tobacco Survey, provide valuable opportunities to examine adolescent behavioural risk factors at the population level. Previous national studies have reported the prevalence and sociodemographic distribution of tobacco use, dietary behaviours, physical activity, and multiple behavioural risk factors using individual survey waves [11–14] Repeated-survey studies have also examined trends in specific behaviours, including physical activity between 2007 and 2015 [15] and tobacco use between 2009 and 2019 [16]. However, evidence remains fragmented across behaviours, population subgroups, and time. We found no previous study that has harmonised repeated RISKESDAS and SKI surveys to examine long-term trends in multiple adolescent behavioural risk factors while also assessing differences by sex, age group, and geographic region. Consequently, it remains unclear how adolescent behavioural risk patterns have evolved across different population groups and regions in Indonesia. This limitation constrains understanding of changing behavioural risks and the identification of adolescent populations that may require more targeted or sustained prevention efforts.

This knowledge gap is particularly important in Indonesia, where substantial social, economic, and environmental changes have occurred over the past two decades [17]. Rapid urbanisation, digital transformation, and broader socioeconomic changes may all have contributed to shifts in adolescent behavioural trends [18,19]. These changes provide an important context for examining whether adolescent behavioural risk patterns have shifted over time. Understanding how behavioural risk factors vary across population subgroups and change over time may help identify patterns of adolescent health concerns and inform prevention planning and, in turn, resource allocation. Using harmonised nationally representative survey data collected over 16 years, this study provides the first national surveillance analysis of changing patterns of adolescent behavioural risk factors in Indonesia. Specifically, we examined temporal trends in tobacco use, alcohol use, insufficient physical activity, and insufficient fruit and vegetable intake, and assessed how these patterns differed across sex, age group, and geographic region.

## Methods

### Study design

This study used a repeated cross-sectional design based on nationally representative population-based Indonesian health surveys conducted in 2007, 2013, 2018, and 2023. The present analysis focused on adolescents aged 10–18 years sampled within each survey wave. Temporal patterns in tobacco use, alcohol use, physical activity, and fruit and vegetable intake were examined using available comparable behavioural measures across survey waves.

### Data sources

Data were drawn from the RISKESDAS surveys conducted in 2007, 2013, and 2018, and the SKI conducted in 2023 (Table 1). The analysis examined tobacco use, alcohol use, physical activity, and fruit and vegetable intake. These behaviours are core indicators in adolescent health surveillance [14], are closely linked to noncommunicable disease prevention [3], and were available in comparable form across the RISKESDAS and SKI survey waves. Although minor differences existed in questionnaire wording, item structure, or response categories across some survey years, the measures used in the primary analyses were recoded into common binary indicators, with cross-wave differences documented in Supplementary Table S2a. A RISKESDAS survey was also conducted in 2010; however, the dataset was excluded because tobacco use was the only behavioural outcome relevant to the present analysis, and tobacco use data were missing for 59.59% of eligible adolescents (*n* = 24,547 of *N* = 41,192). Given that more than half of the eligible sample lacked tobacco use data and no other study outcomes were available, the remaining observations were considered insufficient for reliable cross-wave comparison.

**Table 1.** Sample characteristics of adolescent participants (*n* = 653,700).

Characteristics	RISKESDAS 2007 (*n* = 173,530)	RISKESDAS 2013 (*n* = 185,631)	RISKESDAS 2018 (*n* = 176,556)	SKI 2023 (*n* = 117,983)	Total
	*n* (%)	*n* (%)	*n* (%)	*n* (%)	*n* (%)
***Sex***					
Male	88,774 (51.16)	95,354 (51.37)	91,403 (51.77)	60,279 (51.09)	335,810 (51.37)
Female	84,756 (48.84)	90,277 (48.63)	85,153 (48.23)	57,704 (48.91)	317,890 (48.63)
***Age group***					
10–12	64,653 (37.26)	69,083 (37.22)	63,815 (36.14)	46,822 (39.69)	244,373 (37.38)
13–15	57,800 (33.31)	63,353 (34.13)	60,557 (34.30)	38,553 (32.68)	220,263 (33.69)
16–18	51,077 (29.43)	53,195 (28.66)	52,184 (29.56)	32,608 (27.64)	189,064 (28.92)
***Region***					
Sumatera	57,610 (33.20)	59,526 (32.07)	54,586 (30.92)	39,487 (33.47)	211,209 (32.31)
Jawa	49,213 (28.36)	50,599 (27.26)	47,215 (26.74)	30,918 (26.21)	177,945 (27.22)
Kepulauan Nusa Tenggara	13,892 (8.01)	16,134 (8.69)	16,379 (9.28)	10,390 (8.81)	56,795 (8.69)
Kalimantan	18,948 (10.92)	18,206 (9.81)	17,456 (9.89)	10,204 (8.65)	64,814 (9.91)
Sulawesi	25,790 (14.86)	26,157 (14.09)	26,731 (15.14)	18,950 (16.06)	97,628 (14.93)
Kepulauan Maluku	3,963 (2.28)	7,223 (3.89)	7,206 (4.08)	4,343 (3.68)	22,735 (3.48)
Papua	4,114 (2.37)	7,786 (4.19)	6,983 (3.96)	3,691 (3.13)	22,574 (3.45)

Note: Data are presented as unweighted. Province classifications differed slightly across survey waves and were harmonised into broader geographic regions.

RISKESDAS and SKI are nationally representative health surveys administered by the Indonesian Ministry of Health as part of routine national health surveillance. Both data sets were collected using standardised interviewer-administered household and individual questionnaires covering demographic characteristics, health conditions, and behavioural risk factors. The original survey instruments recorded respondent mode at the individual level, including direct interviews, interviews with assistance or accompaniment, and, in some waves, responses by representation; adolescents younger than 15 years were interviewed with an accompanying household member. However, these respondent-mode variables were not available in the released datasets, so we could not determine the proportion interviewed by each mode or assess whether respondent mode differed across age groups or survey waves. All survey waves employed multistage stratified sampling procedures to ensure representation across provinces in Indonesia. Sampling involved staged selection of census blocks followed by systematic household selection within sampled census blocks. The surveys were designed to support national and subnational health monitoring and have been widely used in epidemiological and public health research in Indonesia [11,12].

### Participants

The analytic sample comprised 653,700 adolescents aged 10–18 years across the 2007, 2013, 2018, and 2023 surveys. Sample sizes are unweighted counts; survey weights were used only for population-level estimation. No data were missing for sex, age group, or geographic region, and no participants were excluded for missing demographic information. For available behavioural outcomes, source variables and questionnaire skip patterns were checked, with no item-level missing data identified. Apparent missing values in conditional follow-up items were structural skips and were coded according to the preceding screening response; therefore, no participants were excluded for missing outcome data. Alcohol use and insufficient fruit and vegetable intake were unavailable in the released 2013 dataset and were not analysed for that wave. Characteristics of participants in each survey wave are presented in Table 1.

### Measures

Behavioural risk factors were selected based on the availability of comparable measures across the four survey waves. The analysis included current tobacco use, current alcohol use, insufficient physical activity, and insufficient fruit and vegetable intake. Some question wording, response structures, and item formats varied across survey waves; hence, the variables were recoded into standardised binary indicators. Details of survey items and response categories across survey waves are provided in Supplementary Table S2a and Table S2b. Each behavioural risk factor was coded dichotomously, with 1 indicating the presence of the risk factor and 0 indicating its absence.

#### Tobacco use

Tobacco use was assessed using smoking and chewing tobacco items referring to use in the past 30 days. In the 2007 survey, smoking and chewing tobacco were assessed using a combined item, whereas the 2013, 2018, and 2023 surveys assessed smoking and chewing tobacco separately before harmonisation into a single binary indicator. Current tobacco use was defined as smoking and/or chewing tobacco on at least one day in the past 30 days [20]. Because smoking and chewing tobacco could not be separated in the 2007 survey, component-specific analyses of current smoking and current chewing tobacco were restricted to 2013, 2018, and 2023.

#### Alcohol use

Alcohol use was assessed using an alcohol consumption item referring to use in the past 30 days. Current alcohol use was defined as consuming at least one alcoholic drink in the past 30 days [21]. A comparable alcohol measure was available in the 2007, 2018, and 2023 survey waves, whereas the corresponding 2013 variable was unavailable in the released dataset.

#### Physical activity

Physical activity was assessed using vigorous- and moderate-intensity physical activity items capturing usual participation in activities performed continuously for at least 10 minutes at a time. Duration (minutes per day) and frequency (days per week) of vigorous- and moderate-intensity activities were collected separately. For each intensity, minutes spent undertaking physical activity per week were calculated by multiplying the reported duration in minutes per day by the reported number of days per week. Weekly vigorous- and moderate-intensity activity minutes were then summed to derive total weekly moderate-to-vigorous physical activity (MVPA), and average daily MVPA was calculated by dividing this total by 7. Insufficient physical activity was defined as engaging in less than 60 minutes of MVPA per day [22]. The same harmonised derivation algorithm was applied across all survey waves, with source-variable coding and skip patterns verified against the original survey questionnaires.

#### Fruit and vegetable intake

Fruit intake was calculated by multiplying the reported number of fruit-consumption days per week by the number of fruit portions consumed per consumption day. The same calculation was applied to vegetables. The resulting weekly fruit and vegetable estimates were summed and divided by 7 to derive the estimated average combined intake per day. Insufficient fruit and vegetable intake was defined as fewer than five portions per day, based on the recommended intake for adolescents aged 10 years and older [23]. Exact equivalence of the visual aids used to estimate portion size across survey waves could not be verified. Although the 2013 survey instrument included fruit and vegetable intake items, the corresponding variables were unavailable in the released dataset and were excluded from corresponding analyses.

#### Sociodemographic variables

Sociodemographic variables included sex, age group, and geographic region, which were comparable across the survey waves. Socioeconomic indicators such as household income, parental education, or comparable wealth measures were not available in the released survey datasets and therefore could not be included in the analyses. Sex was categorised as male or female. Age was grouped into three categories representing early, middle, and late adolescence: 10–12 years, 13–15 years, and 16–18 years [24]. Province codes were harmonised across survey waves and aggregated into seven major geographic regions: Sumatera, Jawa, Kepulauan Nusa Tenggara, Kalimantan, Sulawesi, Kepulauan Maluku, and Papua. Province-to-region mappings are provided in Supplementary Table S2a.

### Statistical analysis

All analyses accounted for the complex survey design of each survey wave by incorporating sampling weights, strata, and primary sampling units. Before pooling, survey-specific primary sampling unit and stratum identifiers were recoded to ensure that they were unique across survey years [25], and singleton strata were addressed using centred variance estimation. Original sampling weights were retained in the primary pooled analyses, such that survey waves contributed according to their original weight totals. Because the relative scaling of survey weights should be considered when combining survey cycles [26], weights were rescaled within each survey wave to a common total in a sensitivity analysis to assess whether the adjusted estimates were sensitive to the relative weighting of survey cycles. Survey-weighted prevalence estimates with 95% confidence intervals (CIs) were calculated for each behavioural risk factor overall and by sex, age group, and geographic region. These estimates represented the observed population prevalence in each survey wave.

Separate survey-weighted Poisson regression models with robust standard errors were used to estimate adjusted prevalence ratios (aPRs) and 95% CIs. This approach provided interpretable prevalence ratios and avoided the overestimation that can arise from odds ratios when outcomes are highly prevalent [27]. Survey year was modelled categorically using the earliest available year as the reference, with adjustment for sex, age group, and geographic region. Whereas the survey-weighted prevalence estimates represented the observed population prevalence in each survey wave, the aPRs estimated relative differences between survey years after accounting for differences in sex, age-group, and geographic composition. Consequently, adjusted temporal estimates could differ in magnitude or direction from the observed prevalence patterns. Marginal adjusted prevalence estimates were also derived for each survey year to present these adjusted temporal differences on the prevalence scale and facilitate interpretation alongside the observed prevalence estimates.

Differences in temporal patterns across population subgroups were assessed using separate year-by-sex, year-by-age group, and year-by-region interaction models, with each set of interaction terms evaluated using a joint Wald test. No multiplicity correction was applied because the subgroup and interaction analyses were intended to describe heterogeneity in temporal patterns. Given the large sample size and number of comparisons, interpretation emphasised the magnitude and precision of prevalence differences and their public health relevance rather than statistical significance alone. These models assessed effect modification separately and did not examine intersections between multiple sociodemographic characteristics. Separate subgroup-stratified models were then fitted to describe adjusted temporal patterns within each sex, age group, and geographic region. Sex-stratified models were adjusted for age group and geographic region; age-group-stratified models were adjusted for sex and geographic region; and region-stratified models were adjusted for sex and age group.

Two additional sensitivity analyses were conducted to assess the robustness of the main findings. First, physical activity models were repeated after excluding 2013 to assess whether the findings were dependent on this survey wave. Second, for tobacco use, separate prevalence estimates for smoking and chewing tobacco were calculated for 2013–2023, and the regression and interaction analyses were repeated using smoking alone. All analyses were conducted using Stata (version 19.0; StataCorp LLC, College Station, TX, USA).

## Results

### Participant characteristics

A total of 653,700 adolescents aged 10–18 years were included across the four survey waves; specifically, 173,530 (2007), 185,631 (2013), 176,556 (2018), and 117,983 (2023). Based on the unweighted distributions, sex was relatively balanced in each survey year, with males comprising 51.09%–51.77% of participants. Adolescents aged 10–12 years consistently represented the largest age group across survey waves (36.14%–39.69%), whereas adolescents aged 16–18 years represented the smallest proportion (27.64%–29.56%). Geographically, the largest proportion of participants was drawn from Sumatera (30.92%–33.47%) and Jawa (26.21%–28.36%) across all survey years, while Kepulauan Maluku (2.28%–4.08%) and Papua (2.37%–4.19%) contributed the smallest proportions of the analytic sample (see Table 1).

### Overall temporal patterns

Overall prevalence estimates are presented in Figure 1. Survey-weighted tobacco use prevalence increased from 7.84% (95% CI 7.67–8.00) in 2007 to 10.08% (9.84–10.31) in 2018, an increase of 2.24% points, before declining to 8.19% (7.87–8.51) in 2023. In smoking-only sensitivity analyses (see Supplementary Table S11) restricted to 2013–2023, smoking prevalence was higher in 2018 than in 2013 (aPR 1.15, 95% CI 1.11–1.18) and lower in 2023 (0.92, 0.88–0.96). Alcohol use prevalence was broadly comparable in 2007 (1.77%, 95% CI 1.69–1.85) and 2018 (1.63%, 1.54–1.72) but was substantially lower in 2023 (0.72%, 0.64–0.80), 1.05% points below the 2007 prevalence. The estimated prevalence of insufficient physical activity was markedly lower in the 2013 survey (45.90%, 95% CI 45.26–46.53) than in 2007 (66.96%, 66.55–67.37), a difference of 21.06% points, before returning to 66.17% (65.72–66.61) in 2018 and declining to 61.78% (61.13–62.43) in 2023. In sensitivity analyses excluding the 2013 survey (see Supplementary Table S9), insufficient physical activity remained similar in 2018 compared with 2007 (1.005, 0.996–1.014) and lower in 2023 (0.939, 0.928–0.950). Temporal variation by sex, age group, and region was retained (all *p* < 0.001). Thus, although prevalence rebounded after 2013, it remained 5.18% points below its 2007 level in 2023. Insufficient fruit and vegetable intake remained high, changing from 79.34% (78.96–79.72) in 2007 to 78.27% (77.92–78.63) in 2018 and increasing to 84.47% (84.07–84.88) in 2023, 5.13% points above the 2007 prevalence. Data on alcohol use and fruit and vegetable intake were unavailable in the released 2013 dataset.

**Figure 1.**
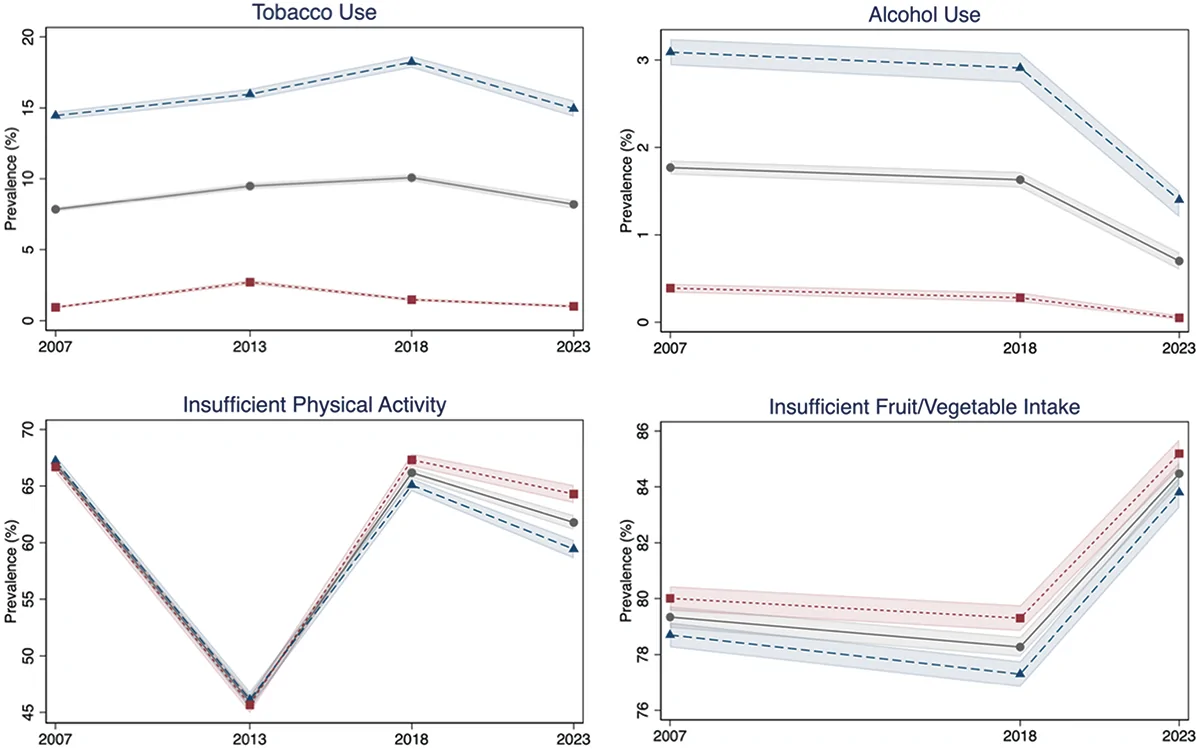
Temporal trends in behavioural risk factors overall and by sex among Indonesian adolescents, 2007–2023.

Note: Survey-weighted prevalence (%) with shaded area represents 95% CIs, ––––––––– Overall, — — — — Male, and – – – – – Female. Tobacco use includes smoking and/or chewing tobacco. Alcohol use and insufficient fruit and vegetable intake data were unavailable in the released 2013 dataset. Detailed numerical estimates are provided in Supplementary Table S3.

### Differences by sex

Prevalence estimates by sex are presented in Figure 1. Tobacco and alcohol use were consistently more prevalent among males than females, whereas insufficient physical activity and insufficient fruit and vegetable intake were generally more prevalent among females. Among males, tobacco use prevalence increased from 14.46% (14.16–14.75) in 2007 to 18.23% (17.82–18.65) in 2018, before declining to 14.95% (14.38–15.53) in 2023. Among females, component-specific estimates from 2013 onward showed that combined tobacco prevalence was predominantly attributable to chewing tobacco rather than smoking; female smoking prevalence remained below 0.31% in all three available waves (Supplementary Table S10). Alcohol use prevalence was also consistently higher among males, with a prevalence of 3.09% (2.94–3.24) in 2007 and a marked decline by 2023. The prevalence of insufficient physical activity was similar between males and females in 2007 but was higher among females in subsequent survey waves. In 2023, insufficient physical activity affected 64.29% (63.49–65.08) of females compared with 59.43% (58.61–60.24) of males. Insufficient fruit and vegetable intake was also consistently higher among females and reached its highest prevalence in 2023 among both males (83.80%, 83.25–84.35) and females (85.19%, 84.66–85.71).

### Differences by age group

Prevalence estimates by age group are presented in Figure 2. Tobacco and alcohol use were consistently more prevalent among older adolescents, with the highest prevalence observed among adolescents aged 16–18 years. Tobacco use among adolescents aged 16–18 years increased from 19.45% (19.03–19.87) in 2007 to 21.57% (21.02–22.13) in 2018 before declining to 17.96% (17.18–18.75) in 2023. Alcohol use showed a similar pattern, with prevalence consistently highest among adolescents aged 16–18 years. Insufficient physical activity showed an inverse age pattern, with higher prevalence observed among younger adolescents. Adolescents aged 10–12 years showed the highest prevalence, whereas adolescents aged 16–18 years consistently showed the lowest prevalence. Insufficient fruit and vegetable intake remained high across all age groups and was highest in 2023 across all age categories.

**Figure 2.**
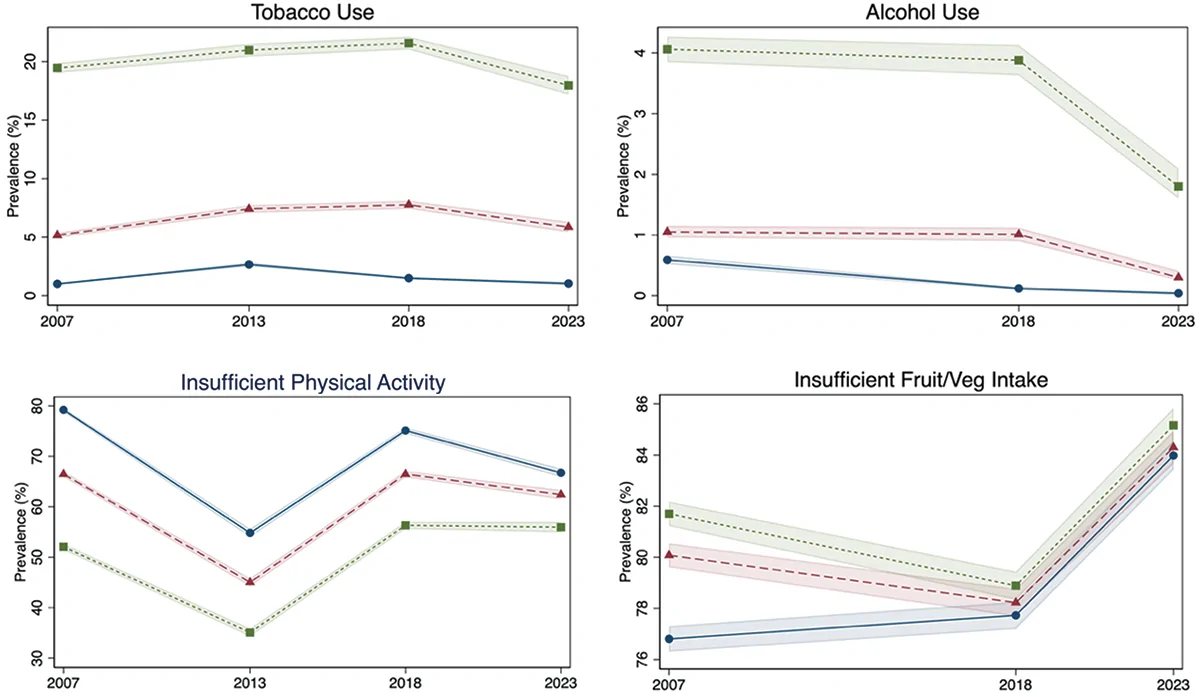
Temporal trends in behavioural risk factors by age group among Indonesian adolescents, 2007–2023.

Note: Survey-weighted prevalence (%) with shaded area represents 95% CIs, 10–12 years,  13–15 years, and 16–18 years. Tobacco use includes smoking and/or chewing tobacco. Alcohol use and insufficient fruit and vegetable intake data were unavailable in the released 2013 dataset. Detailed numerical estimates are provided in Supplementary Table S3.

### Regional differences

Regional prevalence estimates are presented in Figure 3. Papua had the highest combined tobacco prevalence from 2013 onward. Component-specific estimates showed that this pattern was largely attributable to chewing tobacco rather than smoking (Supplementary Table S10). Kalimantan showed the lowest combined tobacco prevalence at 5.13% (4.51–5.75). Alcohol use prevalence was consistently highest in Kepulauan Nusa Tenggara, peaking at 4.73% (4.26–5.21) in 2018, while prevalence remained comparatively lower in Sumatera, Jawa, and Kalimantan. Physical inactivity prevalence remained high across all regions throughout the study period, although regional patterns differed over time. Similarly, insufficient fruit and vegetable intake was consistently high across all regions and was highest in 2023 across most geographic regions.

**Figure 3.**
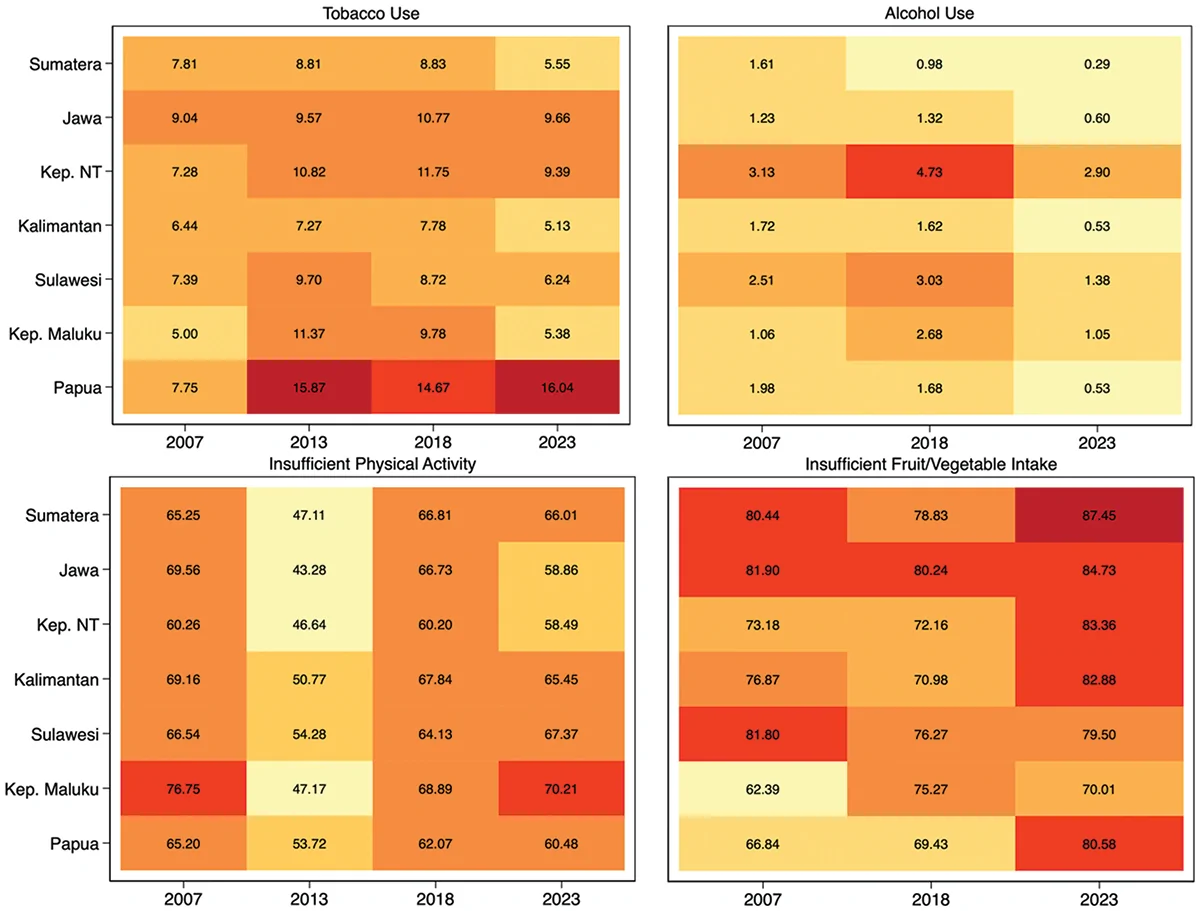
Temporal trends in behavioural risk factors across geographic regions among Indonesian adolescents, 2007–2023.

Note: Heat map colours represent prevalence intensity, with darker shades indicating higher prevalence. Numeric values within cells represent survey-weighted prevalence estimates (%). Kep. NT = Kepulauan Nusa Tenggara. Tobacco use includes smoking and/or chewing tobacco. Alcohol use and insufficient fruit and vegetable intake data were unavailable in the released 2013 dataset. Detailed numerical estimates are provided in Supplementary Table S4.

### Adjusted temporal comparisons

After adjustment for sex, age group, and geographic region, tobacco use prevalence was higher in 2013 (aPR 1.17, 95% CI 1.14–1.21) and 2018 (1.15, 1.12–1.19) than in 2007, but lower in 2023 (0.93, 0.89–0.96; Table 2). Temporal patterns varied across sex age group and geographic region, athough the magnitude and public health relevance of these differences varied across subgroups (interaction tests all *p* < 0.001; Supplementary Table S5). Among males, prevalence declined below its 2007 level by 2023 (0.90, 0.86–0.93), whereas it remained higher among females (1.22, 1.07–1.41). However, separate analyses from 2013 onward showed that chewing tobacco contributed substantially to the combined tobacco estimates among females. The 2023 reduction was also confined to adolescents aged 16–18 years (0.86, 0.83–0.90). Regionally, tobacco use prevalence remained higher than in 2007 in Papua (1.94, 1.57–2.40) and Kepulauan Nusa Tenggara (1.19, 1.06–1.33), but was lower in Sumatera, Kalimantan, and Sulawesi. In smoking-only sensitivity analyses restricted to 2013–2023, temporal variation remained evident across sex, age group, and region, although the sex-specific variation was considerably weaker (*p* = 0.044 for sex and *p* < 0.001 for age group and region; Supplementary Table S11).

**Table 2.** Differences in adolescent behavioural risk factors across survey years after accounting for sex, age, and region, 2007–2023.

Survey year	aPR (95% CI)	p-value
*Tobacco use*		
2007 (Reference)	1	
2013	1.17 (1.14–1.21)	<0.001
2018	1.15 (1.12–1.19)	<0.001
2023	0.93 (0.89–0.96)	<0.001
*Alcohol use*		
2007 (Reference)	1	
2018	0.95 (0.88–1.02)	0.128
2023	0.41 (0.37–0.46)	<0.001
*Insufficient physical activity*		
2007 (Reference)	1	
2013	0.70 (0.68–0.71)	<0.001
2018	1.01 (1.00–1.02)	0.008
2023	0.95 (0.94–0.96)	<0.001
*Insufficient fruit and vegetable intake*		
2007 (Reference)	1	
2018	0.98 (0.97–0.98)	<0.001
2023	1.05 (1.05–1.06)	<0.001

Note: aPR = adjusted prevalence ratio. Estimates derived from adjusted survey-weighted Poisson regression models with robust standard errors. The earliest available survey year for each behavioural outcome was the reference category.

Adjusted alcohol use prevalence in 2018 was similar to that in 2007, whereas substantially lower prevalence was observed in 2023 (aPR 0.41, 95% CI 0.37–0.46). Temporal patterns varied across sex, age group, and geographic region (interaction tests all *p* < 0.001). In 2018, prevalence remained similar to 2007 among males (0.98, 0.91–1.05) but was lower among females (0.67, 0.53–0.84) and adolescents aged 10–12 years (0.28, 0.22–0.36). By 2023, prevalence had declined among both sexes, all age groups, and six of the seven regions; no clear difference from 2007 was observed in Kepulauan Maluku.

For insufficient physical activity, adjusted prevalence was substantially lower in 2013 than in 2007 (aPR 0.70, 95% CI 0.68–0.71), while the differences in 2018 and 2023 were comparatively small. Temporal patterns varied across sex, age group, and geographic region (all interaction *p* < 0.001). In 2023, prevalence remained lower than in 2007 among males (0.91, 0.89–0.92) but was similar among females (0.99, 0.97–1.00). Age-specific patterns diverged: prevalence was lower among adolescents aged 10–12 years (0.86, 0.85–0.88) and 13–15 years (0.95, 0.94–0.97), but higher among those aged 16–18 years (1.08, 1.06–1.11). Although adjusted prevalence declined in every region in 2013, patterns subsequently diverged. By 2023, prevalence remained lower than in 2007 in Jawa, Kalimantan, and Kepulauan Maluku, was slightly higher in Sulawesi, and did not differ clearly from 2007 in Sumatera, Kepulauan Nusa Tenggara, or Papua.

Adjusted temporal differences in insufficient fruit and vegetable intake were small overall. Compared with 2007, prevalence was slightly lower in 2018 (aPR 0.98, 95% CI 0.97–0.98) and slightly higher in 2023 (1.05, 1.05–1.06). Temporal variation was evident across age group and geographic region whereas patterns were broadly similar by sex (sex interaction *p* = 0.140). In 2023, prevalence was higher across all age groups, with the largest relative increase among adolescents aged 10–12 years (1.08, 1.07–1.09). Prevalence was also higher in most regions, with the largest relative increases observed in Papua (1.20, 1.13–1.28), Kepulauan Nusa Tenggara (1.14, 1.11–1.17), and Kepulauan Maluku (1.12, 1.05–1.19). Sulawesi was the only region with slightly lower prevalence than in 2007 (0.97, 0.96–0.99). Subgroup-specific adjusted estimates are presented in Supplementary Tables S6–S8.

Sensitivity analyses using survey-cycle-normalised (rescaled survey) weights produced materially similar adjusted temporal estimates across all four behavioural outcomes (Supplementary Table S12). The largest difference was observed for insufficient physical activity in 2018, for which the estimate moved closer to the null (1.007, 0.998–1.016). Marginal adjusted prevalence estimates showed patterns corresponding to the aPRs, including lower adjusted tobacco prevalence in 2023 than in 2007 and a small increase in adjusted insufficient physical activity prevalence in 2018 despite the opposite direction in the observed prevalence estimates (Supplementary Table S13).

## Discussion

Using nationally representative data collected between 2007 and 2023, this study identified distinct temporal patterns in tobacco use, alcohol use, insufficient physical activity, and insufficient fruit and vegetable intake among Indonesian adolescents. Survey-weighted tobacco use prevalence peaked in 2018 before returning close to its 2007 level by 2023, although adjusted prevalence in 2023 was lower than in 2007. Alcohol use was broadly comparable in 2007 and 2018 and substantially lower in 2023. In contrast, the marked reduction in insufficient physical activity in 2013 was not sustained, and more than six in ten adolescents remained insufficiently active in 2023. Insufficient fruit and vegetable intake was also persistently high and was 5.13% points higher in 2023 than in 2007. These patterns differed by sex, age group, and geographic region. Tobacco and alcohol use were consistently more prevalent among males and older adolescents, whereas insufficient physical activity was generally higher among females and younger adolescents. Insufficient fruit and vegetable intake was generally higher among females and older adolescents, although its relative increase by 2023 was greatest among the youngest group. These findings extend previous Indonesian studies by showing that multiple behavioural risks followed distinct temporal trends within a harmonised national survey series.

The decline in adolescent alcohol use represents a favourable population-level trend. Prevalence decreased between 2007 and 2023, with most of the reduction occurring after 2018. Adjusted analyses also showed substantially lower prevalence in 2023 than in 2007, and reductions were observed across both sexes, all age groups, and most geographic regions. This pattern is consistent with previous evidence on adolescent alcohol use in Indonesia [28]. Nevertheless, this pattern should be interpreted cautiously. The study cannot determine whether the decline reflects changes in alcohol availability, prevention or regulation, social norms, survey implementation, or other factors. The absence of comparable 2013 data also limits assessment of when the decline began. Despite the overall reduction, alcohol use remained more prevalent among males, older adolescents, and adolescents in Kepulauan Nusa Tenggara, indicating persistent demographic and regional differences.

Insufficient physical activity and insufficient fruit and vegetable intake remained substantial adolescent health concerns. In 2023, more than six in ten adolescents were classified as insufficiently active, and more than eight in ten consumed fewer than five combined portions of fruits and vegetables per day. The markedly lower 2013 estimate of insufficient physical activity should be interpreted cautiously. Re-examination confirmed that the same harmonised derivation algorithm was applied across waves, with no apparent 2013 wave coding or skip-pattern error. The lower estimate was reflected in the underlying responses, including more reported moderate-intensity activity and longer daily duration. However, although the core vigorous- and moderate-intensity questions were highly similar, the broader physical-activity framing differed: the 2007 survey included work, leisure, transport, and separate walking/cycling items; the 2013 survey focused mainly on work and leisure; and the 2018 and 2023 surveys used more detailed activity-recording materials [29–32]. Although these differences were not incorporated into the harmonised MVPA calculation, they may have influenced respondents’ interpretation and reporting of physical activity. Complete measurement equivalence across waves therefore cannot be established.

Interestingly, insufficient physical activity was generally more prevalent among younger than older adolescents, contrasting with many regional and international surveillance studies reporting lower physical activity with increasing age [14,15,33]. These particular findings should therefore be interpreted cautiously because the RISKESDAS and SKI surveys assessed activities performed continuously for at least 10 minutes, which may not fully capture the brief and intermittent movement patterns commonly observed during younger adolescence. One possible explanation is that adolescents often accumulate physical activity through shorter and less structured activities, such as unstructured physical play or transport to and from school [34,35] which may be less likely to be detected using surveillance measures requiring sustained activity periods. Age-stratified models also showed divergent patterns: by 2023, insufficient physical activity remained below 2007 levels among adolescents aged 10–12 and 13–15 years but was higher among those aged 16–18 years. This overall pattern concealed a less favourable change among older adolescents, although cross-wave measurement differences remain relevant.

Tobacco use followed a different temporal pattern. Survey-weighted prevalence increased to 2018 before returning close to its 2007 level by 2023, while adjusted prevalence was lower in 2023 than in 2007. Tobacco use remained substantially more prevalent among males and older adolescents. Greater autonomy, access to tobacco products, peer exposure, and cumulative opportunity for initiation may contribute to the age gradient, although these factors were not measured. Sex-stratified analyses showed that tobacco use prevalence was lower in 2023 than in 2007 among males but remained higher among females. From 2013 onward, female combined tobacco prevalence was driven mainly by chewing tobacco, while female smoking prevalence remained below 0.31% in all three waves. Smoking-only analyses also showed substantially weaker temporal effect modification by sex than the combined tobacco analysis. The larger relative changes among females should be interpreted in light of their very low baseline prevalence and the contribution of chewing tobacco, as small absolute changes can produce large relative differences. Reported tobacco use may also be affected by social desirability bias, particularly among females where smoking may be less socially acceptable [16]. Prevention should therefore continue to prioritise males because of their substantially higher absolute prevalence, while surveillance among females remains warranted given differing temporal patterns of smoking and chewing tobacco. Similar subgroup patterns reported elsewhere may reflect gender norms, peer influences, and the social acceptability of substance use [7,36], while tobacco and alcohol advertising near schools may also contribute [37]. These factors were not directly measured and should therefore be regarded as possible explanations rather than demonstrated causes.

Geographic variation was evident, with relatively higher combined tobacco use prevalence observed in Papua and consistently higher alcohol use prevalence observed in Kepulauan Nusa Tenggara across survey waves. Component-specific analyses for 2013–2023 showed that Papua’s high combined tobacco prevalence was largely attributable to chewing tobacco rather than smoking. In addition to commercial tobacco use, chewing tobacco mixed with areca nut (*pinang*) and lime remains a longstanding sociocultural practice across many Melanesian and Papuan communities [38]. The high combined tobacco prevalence observed in Papua may therefore partly reflect the prominence of chewing tobacco within local sociocultural practices. Other contextual factors, including health education and tobacco control regulations, may also contribute [37,39–41], although these were not measured in the present study. Similarly, traditional alcoholic beverages such as *sopi* and *moke* remain embedded within customary rituals and social ceremonies in several communities in Kepulauan Nusa Tenggara [42,43]. Although sociocultural determinants were not directly assessed, regional variation in adolescent substance use may reflect differences in local norms, traditional practices, and public health regulation, highlighting the need for prevention approaches sensitive to regional contexts rather than relying solely on standardised national strategies.

### Implications

Taken together, these findings have several implications for adolescent health monitoring and prevention strategies in Indonesia. Importantly, the public health significance of these findings lies less in the statistical significance of individual temporal contrasts than in the substantial absolute burden of some behaviours and the persistence of marked demographic and regional differences. First, tobacco and alcohol use may require targeted prevention efforts for males, older adolescents, and regions with higher observed prevalence. For tobacco, prevention and surveillance should distinguish between smoked and chewing forms, particularly in regions such as Papua where the high combined prevalence was largely attributable to chewing tobacco. Such approaches should be informed by local evidence regarding substance availability, marketing exposure, social norms, and regulatory implementation rather than assuming that the same explanation or strategy applies across regions [16,39]. Second, insufficient physical activity and insufficient fruit and vegetable intake were widespread across demographic and geographic groups. These behaviours may therefore require broad strategies delivered through schools, families, and communities [3]. Potential priorities include increasing opportunities for physical activity during the school day, supporting active travel and recreation, improving access to affordable fruits and vegetables, and strengthening food environments around schools. Intervention development should account for adolescents’ daily routines and the practical constraints operating across home, school, and community settings.

### Strengths and limitations

This study has several important strengths. First, the analysis used large nationally representative health surveys spanning a 16-year period, allowing examination of long-term temporal patterns in adolescent behavioural risk factors in Indonesia. The pooled sample of more than 650,000 adolescents provided substantial statistical power and enabled subgroup analyses across sex, age group, and geographic region. Second, the study examined multiple behavioural risk factors simultaneously using harmonised behavioural measures across survey waves, providing a broader understanding of adolescent behavioural health patterns. Third, the use of survey-weighted analyses accounted for the complex sampling design of the national surveys and improved the representativeness of prevalence estimates at the population level. Finally, the inclusion of regional analyses strengthened the policy relevance of the study by identifying regions where specific behavioural risk factors were more concentrated, supporting more locally responsive adolescent health strategies in Indonesia.

Several limitations should be considered when interpreting the findings of this study. Differences in questionnaire structure, item wording, and response categories (e.g. variations related to smoking and smokeless tobacco use) across survey waves may also have affected temporal comparability despite efforts to standardise behavioural measures across surveys. In 2007, smoking and chewing tobacco were assessed using a combined item, but separately from 2013 onward; their individual contributions therefore could not be examined across the full 2007–2023 period. Some behavioural variables were unavailable in specific survey datasets, including alcohol use and fruit and vegetable intake variables in the released 2013 dataset and the exclusion of the 2010 survey because of limited available behavioural variables and substantial missing data. Although the core physical activity items were harmonised, differences in broader domain framing and administration materials may have affected responses; therefore, complete cross-wave measurement equivalence cannot be established. Another limitation is that the analysis did not cover the *Big-6* of adolescent lifestyle risk behaviours. Sedentary behaviour and sleep were not included because comparable measures were not consistently available across the included survey years. Socioeconomic position was not modelled, as the released analytic datasets did not include income or a comparable set of household asset and living condition variables from which a wealth index could be constructed. Given established socioeconomic differences in adolescent health behaviours [44], the inability to examine socioeconomic variation limits understanding of whether observed behavioural trends were concentrated among more socioeconomically disadvantaged adolescents. Finally, we could not assess whether respondent mode differed across age groups or survey waves, which may be relevant for socially sensitive behaviours such as tobacco and alcohol use.

## Conclusions

The four behavioural risk factors in Indonesian adolescents showed divergent temporal patterns between 2007 and 2023. Tobacco use returned close to its 2007 level by 2023, while alcohol use declined below its 2007 prevalence. Insufficient physical activity remained high overall, with a markedly lower estimate in the 2013 survey but levels in 2018 and 2023 closer to that observed in 2007, while insufficient fruit and vegetable intake increased over time. These changes were also socially and geographically patterned: tobacco and alcohol use were more prevalent among males and older adolescents, insufficient physical activity was generally more prevalent among females and younger adolescents, and insufficient fruit and vegetable intake was generally more prevalent among females and older adolescents. Regional variation was evident, particularly for combined tobacco use in Papua and alcohol use in Kepulauan Nusa Tenggara. These findings support targeted substance use prevention for higher-prevalence groups and regions, alongside broad strategies through school, family, and community to improve physical activity and fruit and vegetable intake. Continued use of comparable measures in national surveys will be important for determining whether these patterns improve, persist, or worsen.

## Supplementary Material

Clean copy of supplementary data.docx

7 STROBE Checklist.docx

## Data Availability

Researchers may request access to the data through: https://layanandata.kemkes.go.id/katalog-data, subject to approval by the data custodians.
