# Supplementary material for: Divergent temporal patterns in adolescent behavioural risk factors across sex, age, and region in Indonesia: a national repeated cross-sectional study, 2007–2023
Source: Glob Health Action. 2026 Oct 29;19(1):2752484. doi: 10.1080/16549716.2026.2752484 (PMC13721819; doi:10.1080/16549716.2026.2752484)
Supplement: Supplementary file 2 [file ZGHA_A_2752484_SM6036.docx]

**Supplementary Table S1**

*Availability of Behavioural Outcome Data and Analytic Sample Sizes Across Survey Waves, 2007–2023*

| **Variables** | **RISKESDAS** | | | **SKI** | **Total** |
| --- | --- | --- | --- | --- | --- |
|  | **2007** | **2013** | **2018** | **2023** |  |
| Full survey sample (*n*) | 1,048,247 | 1,027,763 | 1,017,290 | 877,531 | 3,970,831 |
| Eligible adolescent sample, aged 10–18 years (*n*) | 173,530 | 185,631 | 176,556 | 117,983 | 653,700 |
| Behavioural outcome availability (*n*) | | | | |  |
| *Tobacco use* | 173,530 | 185,631 | 176,556 | 117,983 | 653,700 |
| *Alcohol consumption* | 173,530 | NA | 176,556 | 117,983 | 468,069 |
| *Physical activity* | 173,530 | 185,631 | 176,556 | 117,983 | 653,700 |
| *Fruit and vegetable intake* | 173,530 | NA | 176,556 | 117,983 | 468,069 |

*Note.* NA = data not available in the released dataset. No item-level missing data were identified for the available behavioural outcomes in any survey wave. Apparent missing values in conditional follow-up items reflected questionnaire skip patterns and were coded according to the preceding screening response; therefore, no participants were excluded from the available outcome analyses because of missing outcome data.

**Supplementary Table S2a**

*Survey Items and Response Categories of Behavioural Risk Factors Across Indonesian National Health Surveys, 2007–2023*

| **Survey items** | **Response categories** |
| --- | --- |
| ***SKI 2023*** | |
| Province | Sumatera  11 Aceh  12 Sumatera Utara  13 Sumatera Barat  14 Riau  15 Jambi  16 Sumatera Selatan  17 Bengkulu  18 Lampung  19 Kepulauan Bangka Belitung  21 Kepulauan Riau  Jawa  31 DKI Jakarta  32 Jawa Barat  33 Jawa Tengah  34 DI Yogyakarta  35 Jawa Timur  36 Banten  Kepulauan Nusa Tenggara  51 Bali  52 Nusa Tenggara Barat  53 Nusa Tenggara Timur  Kalimantan  61 Kalimantan Barat  62 Kalimantan Tengah  63 Kalimantan Selatan  64 Kalimantan Timur  65 Kalimantan Utara  Sulawesi  71 Sulawesi Utara  72 Sulawesi Tengah  73 Sulawesi Selatan  74 Sulawesi Tenggara  75 Gorontalo  76 Sulawesi Barat  Kepulauan Maluku  81 Maluku  82 Maluku Utara  Papua  91 Papua Barat  92 Papua Barat Daya  94 Papua  95 Papua Selatan  96 Papua Tengah  97 Papua Pegunungan |
| Has [NAME] ever smoked? | 1 = Yes, everyday  2 = Yes, not everyday  3 = Never smoked |
| In the past month, has [NAME] smoked? | 1 = Yes, everyday  2 = Yes, not everyday  3 = I quit smoking |
| Has [NAME] chewed tobacco (e.g., betel quid, betel leaf, or tobacco quid) during the past month? | 1 = Yes, everyday  2 = Yes, sometimes  3 = No, but I have in the past  4 = Never |
| In the past month, has [NAME] consumed any alcoholic beverages? | 1 = Yes  2 = No |
| Does [NAME] usually engage in vigorous-intensity physical activity that is performed continuously for at least 10 minutes each time? | 1 = Yes  2 = No |
| On average, on how many days per week does [NAME] engage in this vigorous-intensity physical activity? | Number of days per week (0–7) |
| On a typical day, how long does [NAME] spend doing this vigorous-intensity physical activity? | Duration per day: hours and minutes (numeric) |
| Does [NAME] usually engage in moderate-intensity physical activity that is performed continuously for at least 10 minutes each time? | 1 = Yes  2 = No |
| On average, on how many days per week does [NAME] engage in this moderate-intensity physical activity? | Number of days per week (0–7) |
| On a typical day, how long does [NAME] spend doing this moderate-intensity physical activity? | Duration per day: hours and minutes (numeric) |
| In a typical week, on how many days does [NAME] eat fresh fruit? | Numeric count of days per week (0–7) |
| On those days, how many portions of fresh fruit does [NAME] usually consume in one day? | Number of portions per day (numeric) |
| In a typical week, on how many days does [NAME] consume vegetables? | Numeric count of days per week (0–7) |
| On those days, how many portions of vegetables does [NAME] usually consume in one day? | Number of portions per day (numeric) |
| ***RISKESDAS 2018*** | |
| Province | Sumatera  11 Aceh  12 Sumatera Utara  13 Sumatera Barat  14 Riau  15 Jambi  16 Sumatera Selatan  17 Bengkulu  18 Lampung  19 Kepulauan Bangka Belitung  21 Kepulauan Riau  Jawa  31 DKI Jakarta  32 Jawa Barat  33 Jawa Tengah  34 DI Yogyakarta  35 Jawa Timur  36 Banten  Kepulauan Nusa Tenggara  51 Bali  52 Nusa Tenggara Barat  53 Nusa Tenggara Timur  Kalimantan  61 Kalimantan Barat  62 Kalimantan Tengah  63 Kalimantan Selatan  64 Kalimantan Timur  65 Kalimantan Utara  Sulawesi  71 Sulawesi Utara  72 Sulawesi Tengah  73 Sulawesi Selatan  74 Sulawesi Tenggara  75 Gorontalo  76 Sulawesi Barat  Kepulauan Maluku  81 Maluku  82 Maluku Utara  Papua  91 Papua Barat  94 Papua |
| Has [NAME] ever smoked? | 1 = Yes, everyday  2 = Yes, not everyday  3 = Never smoked |
| In the past month, has [NAME] smoked? | 1 = Yes, everyday  2 = Yes, not everyday  3 = I quit smoking |
| Has [NAME] chewed tobacco (e.g., betel quid, betel leaf, or tobacco quid) during the past month? | 1 = Yes, everyday  2 = Yes, sometimes  3 = No, but I have in the past  4 = Never |
| In the past month, has [NAME] consumed any alcoholic beverages? | 1 = Yes  2 = No |
| Does [NAME] usually engage in vigorous-intensity physical activity that is performed continuously for at least 10 minutes each time? | 1 = Yes  2 = No |
| On average, on how many days per week does [NAME] engage in this vigorous-intensity physical activity? | Number of days per week (0–7) |
| On a typical day, how long does [NAME] spend doing this vigorous-intensity physical activity? | Duration per day: hours and minutes (numeric) |
| Does [NAME] usually engage in moderate-intensity physical activity that is performed continuously for at least 10 minutes each time? | 1 = Yes  2 = No |
| On average, on how many days per week does [NAME] engage in this moderate-intensity physical activity? | Number of days per week (0–7) |
| On a typical day, how long does [NAME] spend doing this moderate-intensity physical activity? | Duration per day: hours and minutes (numeric) |
| In a typical week, on how many days does [NAME] eat fresh fruit? | Number of days per week (0–7) |
| On those days, how many portions of fresh fruit does [NAME] usually consume in one day? | Number of portions per day (numeric) |
| In a typical week, on how many days does [NAME] consume vegetables? | Number of days per week (0–7) |
| On those days, how many portions of vegetables does [NAME] usually consume in one day? | Number of portions per day (numeric) |
| ***RISKESDAS 2013*** | |
| Province | Sumatera  11 Aceh  12 Sumatera Utara  13 Sumatera Barat  14 Riau  15 Jambi  16 Sumatera Selatan  17 Bengkulu  18 Lampung  19 Kepulauan Bangka Belitung  21 Kepulauan Riau  Jawa  31 DKI Jakarta  32 Jawa Barat  33 Jawa Tengah  34 DI Yogyakarta  35 Jawa Timur  36 Banten  Kepulauan Nusa Tenggara  51 Bali  52 Nusa Tenggara Barat  53 Nusa Tenggara Timur  Kalimantan  61 Kalimantan Barat  62 Kalimantan Tengah  63 Kalimantan Selatan  64 Kalimantan Timur  Sulawesi  71 Sulawesi Utara  72 Sulawesi Tengah  73 Sulawesi Selatan  74 Sulawesi Tenggara  75 Gorontalo  76 Sulawesi Barat  Kepulauan Maluku  81 Maluku  82 Maluku Utara  Papua  91 Papua Barat  94 Papua |
| In the past month, has [NAME] smoked? | 1 = Yes, daily smoker  2 = Yes, occasional smoker  3 = No, former daily smoker  4 = No, former occasional smoker  5 = Never smoked |
| Has [NAME] chewed tobacco (e.g., betel quid, betel leaf, or tobacco quid) during the past month? | 1 = Yes, daily  2 = Yes, occasional  3 = No, former daily  4 = No, former occasional  5 = Never |
| Does [NAME] usually engage in vigorous-intensity physical activity that is performed continuously for at least 10 minutes each time? | 1 = Yes  2 = No |
| On average, on how many days per week does [NAME] engage in this vigorous-intensity physical activity? | Number of days per week (0–7) |
| On a typical day, how long does [NAME] spend doing this vigorous-intensity physical activity? | Duration per day: hours and minutes (numeric) |
| Does [NAME] usually engage in moderate-intensity physical activity that is performed continuously for at least 10 minutes each time? | 1 = Yes  2 = No |
| On average, on how many days per week does [NAME] engage in this moderate-intensity physical activity? | Number of days per week (0–7) |
| On a typical day, how long does [NAME] spend doing this moderate-intensity physical activity? | Duration per day: hours and minutes (numeric) |
| In a typical week, on how many days does [NAME] eat fresh fruit? | Numeric count of days per week (0–7) |
| On those days, how many portions of fresh fruit does [NAME] usually consume in one day? | Number of portions per day (numeric) |
| In a typical week, on how many days does [NAME] consume vegetables? | Numeric count of days per week (0–7) |
| On those days, how many portions of vegetables does [NAME] usually consume in one day? | Number of portions per day (numeric) |
| ***RISKESDAS 2007*** | |
| Province | Sumatera  11 Aceh  12 Sumatera Utara  13 Sumatera Barat  14 Riau  15 Jambi  16 Sumatera Selatan  17 Bengkulu  18 Lampung  19 Kepulauan Bangka Belitung  21 Kepulauan Riau  Jawa  31 DKI Jakarta  32 Jawa Barat  33 Jawa Tengah  34 DI Yogyakarta  35 Jawa Timur  36 Banten  Kepulauan Nusa Tenggara  51 Bali  52 Nusa Tenggara Barat  53 Nusa Tenggara Timur  Kalimantan  61 Kalimantan Barat  62 Kalimantan Tengah  63 Kalimantan Selatan  64 Kalimantan Timur  Sulawesi  71 Sulawesi Utara  72 Sulawesi Tengah  73 Sulawesi Selatan  74 Sulawesi Tenggara  75 Gorontalo  76 Sulawesi Barat  Kepulauan Maluku  81 Maluku  82 Maluku Utara  Papua  91 Papua Barat  94 Papua |
| In the past month, has [NAME] smoked or chewed tobacco? | 1 = Yes, daily  2 = Yes, occasionally  3 = No, but previously smoked  4 = Never smoked at all |
| In the past month, has [NAME] consumed any alcoholic beverages? | 1 = Yes  2 = No |
| Does [NAME] usually engage in vigorous-intensity physical activity that is performed continuously for at least 10 minutes each time? | 1 = Yes  2 = No |
| On average, on how many days per week does [NAME] engage in this vigorous-intensity physical activity? | Number of days per week (0–7) |
| On a typical day, how long does [NAME] spend doing this vigorous-intensity physical activity? | Duration per day: hours and minutes (numeric) |
| Does [NAME] usually engage in moderate-intensity physical activity that is performed continuously for at least 10 minutes each time? | 1 = Yes  2 = No |
| On average, on how many days per week does [NAME] engage in this moderate-intensity physical activity? | Number of days per week (0–7) |
| On a typical day, how long does [NAME] spend doing this moderate-intensity physical activity? | Duration per day: hours and minutes (numeric) |
| In a typical week, on how many days does [NAME] eat fresh fruit? | Numeric count of days per week (0–7) |
| On those days, how many portions of fresh fruit does [NAME] usually consume in one day? | Number of portions per day (numeric) |
| In a typical week, on how many days does [NAME] consume vegetables? | Numeric count of days per week (0–7) |
| On those days, how many portions of vegetables does [NAME] usually consume in one day? | Number of portions per day (numeric) |

*Note*. Province codes were harmonised into seven major Indonesian islands classification. Tobacco use indicators were harmonised across survey years despite differences in smoking and chewing tobacco item structures. Physical activity items assessed moderate- and vigorous-intensity activities performed continuously for at least 10 minutes. Fruit and vegetable intake items were used to derive average daily consumption estimates. Physical activity items assessed vigorous- and moderate-intensity activities performed continuously for at least 10 minutes. The core frequency and duration items were similar across waves, although broader domain framing, additional physical-activity items, and supporting administration materials differed between surveys. These additional items were not included in the harmonised MVPA derivation.

**Supplementary Table S2b.**

*Details of Survey Items, Response Categories, and Harmonised Coding Across Survey Waves*

| **Outcome** | **Survey wave** | **Harmonised coding/derivation** |
| --- | --- | --- |
| Tobacco use | 2007 | Responses 1 (Yes, daily) and 2 (Yes, occasionally) coded as current tobacco use = 1; responses 3–4 coded = 0. |
| Tobacco use | 2013 | Smoking responses 1–2 coded as current smoking = 1 and 3–5 = 0; chewing responses 1–2 coded as current chewing = 1 and 3–5 = 0. Current tobacco use = 1 if either smoking or chewing = 1. |
| Tobacco use | 2018 | Past-month smoking responses 1–2 coded = 1 and response 3 = 0; respondents who had never smoked were coded 0. Chewing responses 1–2 coded = 1 and 3–4 = 0. Current tobacco use = 1 if either smoking or chewing = 1. |
| Tobacco use | 2023 | Same harmonised coding as 2018. |
| Alcohol use | 2007, 2018, 2023 | Response 1 (Yes) coded as current alcohol use = 1; response 2 (No) coded = 0. |
| Physical activity | All waves | Vigorous and moderate activity were derived separately as days/week × duration/day. Weekly vigorous and moderate minutes were summed and divided by 7; <60 min/day coded as insufficient physical activity = 1 and ≥60 min/day = 0. |
| Fruit and vegetable intake | 2007, 2018, 2023 | Weekly fruit intake = fruit days/week × portions/day; weekly vegetable intake calculated similarly. Combined weekly intake was divided by 7; <5 portions/day coded as insufficient intake = 1 and ≥5 portions/day = 0. |

**Supplementary Table S3**

*Overall Prevalence of Behavioural Risk Factors among Indonesian Adolescents and Stratified Estimates by Sex and Age Group, 2007–2023*

| **Survey Year** | **Overall, % (95% CI)** | **Sex, % (95% CI)** | | **Age, % (95% CI)** | | |
| --- | --- | --- | --- | --- | --- | --- |
|  |  | **Male** | **Female** | **10–12** | **13–15** | **16–18** |
| *Tobacco use* | | | | | | |
| 2007 | 7.84 (7.67–8.00) | 14.46 (14.16–14.75) | 0.94 (0.85–1.03) | 1.01 (0.92–1.11) | 5.16 (4.94–5.38) | 19.45 (19.03–19.87) |
| 2013 | 9.49 (9.27–9.71) | 15.97 (15.59–16.35) | 2.71 (2.55–2.87) | 2.67 (2.49–2.85) | 7.41 (7.08–7.73) | 20.97 (20.41–21.52) |
| 2018 | 10.08 (9.84–10.31) | 18.23 (17.82–18.65) | 1.48 (1.37–1.59) | 1.50 (1.37–1.62) | 7.77 (7.42–8.12) | 21.57 (21.02–22.13) |
| 2023 | 8.19 (7.87–8.51) | 14.95 (14.38–15.53) | 1.01 (0.91–1.11) | 1.04 (0.90–1.19) | 5.86 (5.41–6.31) | 17.96 (17.18–18.75) |
| *Alcohol use* | | | | | | |
| 2007 | 1.77 (1.69–1.85) | 3.09 (2.94–3.24) | 0.39 (0.34–0.44) | 0.59 (0.52–0.66) | 1.05 (0.96–1.15) | 4.06 (3.85–4.27) |
| 2018 | 1.63 (1.54–1.72) | 2.91 (2.74–3.08) | 0.28 (0.23–0.34) | 0.12 (0.09–0.14) | 1.01 (0.90–1.12) | 3.88 (3.63–4.13) |
| 2023 | 0.72 (0.64–0.80) | 1.40 (1.20–1.50) | 0.05 (0.03–0.08) | 0.04 (0.01–0.06) | 0.3 (0.25–0.41) | 1.8 (1.6–2.1) |
| *Insufficient physical activity* | | | | | | |
| 2007 | 66.96 (66.55–67.37) | 67.22 (66.73–67.71) | 66.68 (66.19–67.17) | 79.21 (78.72–79.69) | 66.47 (65.92–67.02) | 52.07 (51.46–52.68) |
| 2013 | 45.90 (45.26–46.53) | 46.13 (45.41–46.84) | 45.66 (44.92–46.39) | 54.82 (54.01–55.64) | 45.03 (44.24–45.82) | 35.08 (34.31–35.84) |
| 2018 | 66.17 (65.72–66.61) | 65.08 (64.53–65.64) | 67.30 (66.74–67.86) | 75.11 (74.51–75.70) | 66.48 (65.85–67.12) | 56.32 (55.62–57.01) |
| 2023 | 61.78 (61.13–62.43) | 59.43 (58.61–60.24) | 64.29 (63.49–65.08) | 66.74 (65.90–67.57) | 62.41 (61.49–63.32) | 55.96 (54.95–56.98) |
| *Insufficient fruit and vegetable intake* | | | | | | |
| 2007 | 79.34 (78.96–79.72) | 78.70 (78.26–79.14) | 80.01 (79.57–80.44) | 76.81 (76.31–77.31) | 80.08 (79.61–80.55) | 81.70 (81.22–82.18) |
| 2018 | 78.27 (77.92–78.63) | 77.30 (76.84–77.76) | 79.30 (78.84–79.76) | 77.73 (77.20–78.26) | 78.23 (77.69–78.77) | 78.89 (78.34–79.44) |
| 2023 | 84.47 (84.07–84.88) | 83.80 (83.25–84.35) | 85.19 (84.66–85.71) | 83.98 (83.42–84.55) | 84.30 (83.66–84.94) | 85.16 (84.48–85.84) |

*Note.* Survey-weighted prevalence with 95% confidence interval.

**Supplementary Table S4**

*Regional Prevalence of Behavioural Risk Factors among Indonesian Adolescents, 2007–2023*

| **Survey Year** | **Regions, % (95% CI)** | | | | | | |
| --- | --- | --- | --- | --- | --- | --- | --- |
|  | **Sumatera** | **Jawa** | **Kep. Nusa Tenggara** | **Kalimantan** | **Sulawesi** | **Kep. Maluku** | **Papua** |
| *Tobacco use* | | | | | | | |
| 2007 | 7.81 (7.50–8.12) | 9.04 (8.74–9.33) | 7.28 (6.74–7.82) | 6.44 (6.00–6.88) | 7.39 (6.98–7.80) | 5.00 (4.27–5.72) | 7.75 (6.45–9.04) |
| 2013 | 8.81 (8.47–9.16) | 9.57 (9.22–9.91) | 10.82 (10.06–11.58) | 7.27 (6.70–7.85) | 9.70 (9.18–10.22) | 11.37 (10.13–12.61) | 15.87 (13.66–18.08) |
| 2018 | 8.83 (8.47–9.18) | 10.77 (10.38–11.15) | 11.75 (11.01–12.48) | 7.78 (7.22–8.33) | 8.72 (8.24–9.19) | 9.78 (8.74–10.82) | 14.67 (13.01–16.33) |
| 2023 | 5.55 (5.17–5.93) | 9.66 (9.11–10.21) | 9.39 (8.48–10.30) | 5.13 (4.51–5.75) | 6.24 (5.70–6.78) | 5.38 (4.39–6.38) | 16.04 (13.86–18.22) |
| *Alcohol use* | | | | | | | |
| 2007 | 1.61 (1.47–1.76) | 1.23 (1.11–1.35) | 3.13 (2.78–3.48) | 1.72 (1.45–1.98) | 2.51 (2.26–2.76) | 1.06 (.73–1.38) | 1.98 (1.44–2.51) |
| 2018 | 0.98 (0.86–1.11) | 1.32 (1.18–1.47) | 4.73 (4.26–5.21) | 1.62 (1.33–1.91) | 3.03 (2.75–3.30) | 2.68 (2.03–3.32) | 1.68 (1.26–2.11) |
| 2023 | 0.29 (0.19–0.40) | 0.60 (0.47–0.73) | 2.9 (2.5–3.3) | 0.53 (0.32–0.74) | 1.38 (1.10–1.66) | 1.05 (0.58–1.53) | 0.53 (0.18–0.88) |
| *Insufficient physical activity* | | | | | | | |
| 2007 | 65.25 (64.41–66.08) | 69.56 (68.91–70.20) | 60.26 (58.78–61.73) | 69.16 (68.00–70.34) | 66.54 (65.53–67.56) | 76.75 (74.70–78.80) | 65.20 (62.84–67.56) |
| 2013 | 47.11 (45.94–48.28) | 43.28 (42.28–44.27) | 46.64 (44.84–48.43) | 50.77 (48.91–52.63) | 54.28 (52.80–55.75) | 47.17 (43.87–50.47) | 53.72 (50.59–56.85) |
| 2018 | 66.81 (66.06–67.57) | 66.73 (66.01–67.44) | 60.20 (58.86–61.55) | 67.84 (66.55–69.13) | 64.13 (63.05–65.21) | 68.89 (66.76–71.01) | 62.07 (59.34–64.80) |
| 2023 | 66.01 (64.97–67.04) | 58.86 (57.81–59.92) | 58.49 (56.57–60.40) | 65.45 (63.69–67.21) | 67.37 (66.14–68.60) | 70.21 (67.92–72.51) | 60.48 (56.05–64.92) |
| *Insufficient fruit and vegetable intake* | | | | | | | |
| 2007 | 80.44 (79.75–81.13) | 81.90 (81.32–82.47) | 73.18 (71.50–74.86) | 76.87 (75.58–78.16) | 81.80 (80.93–82.66) | 62.39 (59.41–65.38) | 66.84 (63.76–69.92) |
| 2018 | 78.83 (78.18–79.48) | 80.24 (79.68–80.79) | 72.16 (70.98–73.34) | 70.98 (69.86–72.09) | 76.27 (75.37–77.16) | 75.27 (73.42–77.12) | 69.43 (66.66–72.21) |
| 2023 | 87.45 (86.83–88.07) | 84.73 (84.07–85.38) | 83.36 (82.13–84.59) | 82.88 (81.73–84.02) | 79.50 (78.47–80.53) | 70.01 (67.45–72.57) | 80.58 (77.20–83.96) |

*Note.* Survey-weighted prevalence with 95% confidence interval.

**Supplementary Table S5**

*Survey Year Interactions with Sex, Age Group, and Geographic Region for Behavioural Risk Factors, 2007–2023*

| **Behavioural Outcome** | ***p*-interaction**  **(Year × Sex)** | ***p*-interaction**  **(Year × Age Group)** | ***p*-interaction**  **(Year × Region)** |
| --- | --- | --- | --- |
| Tobacco use | <0.001 | <0.001 | <0.001 |
| Alcohol use | <0.001 | <0.001 | <0.001 |
| Insufficient physical activity | <0.001 | <0.001 | <0.001 |
| Insufficient fruit and vegetable intake | 0.140 | <0.001 | <0.001 |

*Note.* Values are p-values from joint Wald tests of the interaction terms in separate survey-weighted Poisson regression models with robust standard errors. Year-by-sex models were adjusted for age group and geographic region; year-by-age-group models were adjusted for sex and geographic region; and year-by-region models were adjusted for sex and age group.

**Supplementary Table S6**
*Adjusted Temporal Comparisons in Behavioural Risk Factors, Stratified by Sex, among Indonesian Adolescents, 2007–2023*

| **Survey Year** | **Male** | | **Female** | |
| --- | --- | --- | --- | --- |
|  | **aPR (95% CI)** | ***p*-value** | **aPR (95% CI)** | ***p*-value** |
| *Tobacco use* |  |  |  |  |
| 2007 (Reference) | 1 |  | 1 |  |
| 2013 | 1.05 (1.02–1.08) | 0.002 | 3.40 (3.05–3.80) | <0.001 |
| 2018 | 1.11 (1.07–1.14) | <0.001 | 1.83 (1.62–2.07) | <0.001 |
| 2023 | 0.90 (0.86–0.93) | <0.001 | 1.22 (1.07–1.41) | 0.004 |
| *Alcohol use* |  |  |  |  |
| 2007 (Reference) | 1 |  | 1 |  |
| 2018 | 0.98 (0.91–1.05) | 0.569 | 0.67 (0.53–0.84) | 0.001 |
| 2023 | 0.45 (0.40–0.50) | <0.001 | 0.13 (0.08–0.20) | <0.001 |
| *Insufficient physical activity* |  |  |  |  |
| 2007 (Reference) | 1 |  | 1 |  |
| 2013 | 0.70 (0.69–0.71) | <0.001 | 0.69 (0.68–0.71) | <0.001 |
| 2018 | 0.99 (0.98–1.01) | 0.302 | 1.03 (1.02–1.05) | <0.001 |
| 2023 | 0.91 (0.89–0.92) | <0.001 | 0.99 (0.97–1.00) | 0.067 |
| *Insufficient fruit and vegetable intake* |  |  |  |  |
| 2007 (Reference) | 1 |  | 1 |  |
| 2018 | 0.97 (0.97–0.98) | <0.001 | 0.98 (0.97–0.99) | <0.001 |
| 2023 | 1.06 (1.05–1.06) | <0.001 | 1.05 (1.05–1.06) | <0.001 |

*Note.* aPR = adjusted prevalence ratio. Estimates derived from adjusted survey-weighted Poisson regression models with robust standard errors. Overall models were adjusted for sex, age group, and geographic region. Sex-stratified models were adjusted for age group and geographic region. The earliest available survey year for each behavioural outcome was the reference category.

**Supplementary Table S7**
*Adjusted Temporal Comparisons in Behavioural Risk Factors, Stratified by Age Group, among Indonesian Adolescents, 2007–2023*

| **Survey Year** | **10-12** | | **13-15** | | **16-18** | |
| --- | --- | --- | --- | --- | --- | --- |
|  | **aPR (95% CI)** | ***p*-value** | **aPR (95% CI)** | ***p*-value** | **aPR (95% CI)** | ***p*-value** |
| *Tobacco use* |  |  |  |  |  |  |
| 2007 (Reference) | 1 |  | 1 |  | 1 |  |
| 2013 | 2.89 (2.58–3.24) | <0.001 | 1.34 (1.26–1.42) | <0.001 | 1.02 (0.99–1.05) | 0.26 |
| 2018 | 1.63 (1.43–1.85) | <0.001 | 1.41 (1.32–1.49) | <0.001 | 1.04 (1.01–1.07) | 0.006 |
| 2023 | 1.13 (0.96–1.32) | 0.154 | 1.07 (0.98–1.16) | 0.11 | 0.86 (0.83–0.90) | <0.001 |
| *Alcohol use* |  |  |  |  |  |  |
| 2007 (Reference) | 1 |  | 1 |  | 1 |  |
| 2018 | 0.28 (0.22–0.36) | <0.001 | 1.08 (0.95–1.24) | 0.251 | 1.03 (0.96–1.12) | 0.388 |
| 2023 | 0.08 (0.04–0.18) | <0.001 | 0.37 (0.29–0.47) | <0.001 | 0.48 (0.42–0.54) | <0.001 |
| *Insufficient physical activity* |  |  |  |  |  |  |
| 2007 (Reference) | 1 |  | 1 |  | 1 |  |
| 2013 | 0.71 (0.70–0.72) | <0.001 | 0.69 (0.67–0.70) | <0.001 | 0.68 (0.66–0.70) | <0.001 |
| 2018 | 0.97 (0.96–0.98) | <0.001 | 1.01 (1.00–1.03) | 0.025 | 1.09 (1.07–1.11) | <0.001 |
| 2023 | 0.86 (0.85–0.88) | <0.001 | 0.95 (0.94–0.97) | <0.001 | 1.08 (1.06–1.11) | <0.001 |
| *Insufficient fruit and vegetable intake* |  |  |  |  |  |  |
| 2007 (Reference) | 1 |  |  |  | 1 |  |
| 2018 | 1.00 (0.99–1.01) | 0.893 | 0.97 (0.96–0.98) | <0.001 | 0.96 (0.95–0.97) | <0.001 |
| 2023 | 1.08 (1.07–1.09) | <0.001 | 1.04 (1.03–1.05) | <0.001 | 1.04 (1.03–1.05) | <0.001 |

*Note.* aPR = adjusted prevalence ratio. Estimates derived from adjusted survey-weighted Poisson regression models with robust standard errors. Overall models were adjusted for sex, age group, and geographic region. Sex-stratified models were adjusted for sex and geographic region. The earliest available survey year for each behavioural outcome was the reference category.

**Supplementary Table S8**
*Adjusted Temporal Comparisons in Behavioural Risk Factors, Stratified by Geographic Region, among Indonesian Adolescents, 2007–2023*

| **Survey year** | **Sumatera** | | **Jawa** | | **Kep. Nusa Tenggara** | | **Kalimantan** | | **Sulawesi** | | **Kep. Maluku** | | **Papua** | |
| --- | --- | --- | --- | --- | --- | --- | --- | --- | --- | --- | --- | --- | --- | --- |
|  | **aPR (95% CI)** | ***p*-value** | **aPR (95% CI)** | ***p*-value** | **aPR (95% CI)** | ***p*-value** | **aPR (95% CI)** | ***p*-value** | **aPR (95% CI)** | ***p*-value** | **aPR (95% CI)** | ***p*-value** | **aPR (95% CI)** | ***p*-value** |
| *Tobacco use* |  |  |  |  |  |  |  |  |  |  |  |  |  |  |
| 2007 (Reference) | 1 |  | 1 |  | 1 |  | 1 |  | 1 |  | 1 |  | 1 |  |
| 2013 | 1.15 (1.09–1.21) | <0.001 | 1.08 (1.03–1.13) | 0.001 | 1.52 (1.38–1.67) | <0.001 | 1.19 (1.08–1.32) | <0.001 | 1.37 (1.28–1.47) | <0.001 | 2.26 (1.90–2.68) | <0.001 | 2.00 (1.61–2.49) | <0.001 |
| 2018 | 1.11 (1.05–1.17) | <0.001 | 1.11 (1.06–1.15) | <0.001 | 1.53 (1.40–1.67) | <0.001 | 1.14 (1.04–1.25) | 0.004 | 1.10 (1.03–1.18) | 0.008 | 1.87 (1.58–2.21) | <0.001 | 1.83 (1.50–2.24) | <0.001 |
| 2023 | 0.68 (0.63–0.73) | <0.001 | 0.99 (0.94–1.05) | 0.802 | 1.19 (1.06–1.33) | 0.003 | 0.75 (0.66–0.85) | <0.001 | 0.74 (0.68–0.82) | <0.001 | 0.96 (0.77–1.20) | 0.71 | 1.94 (1.57–2.40) | <0.001 |
| *Alcohol use* |  |  |  |  |  |  |  |  |  |  |  |  |  |  |
| 2007 (Reference) | 1 |  | 1 |  | 1 |  | 1 |  | 1 |  | 1 |  | 1 |  |
| 2018 | 0.59 (0.51–0.69) | <0.001 | 0.98 (0.85–1.13) | 0.739 | 1.41 (1.22–1.62) | <0.001 | 0.89 (0.70–1.12) | 0.308 | 1.11 (0.97–1.27) | 0.119 | 2.36 (1.63–3.44) | <0.001 | 0.78 (0.54–1.13) | 0.187 |
| 2023 | 0.17 (0.12–0.25) | <0.001 | 0.44 (0.35–0.55) | <0.001 | 0.81 (0.68–0.98) | 0.027 | 0.29 (0.19–0.44) | <0.001 | 0.47 (0.38–0.59) | <0.001 | 0.82 (0.48–1.40) | 0.458 | 0.24 (0.12–0.48) | <0.001 |
| *Insufficient physical activity* |  |  |  |  |  |  |  |  |  |  |  |  |  |  |
| 2007 (Reference) | 1 |  | 1 |  | 1 |  | 1 |  | 1 |  | 1 |  | 1 |  |
| 2013 | 0.72 (0.70–0.74) | <0.001 | 0.62 (0.61–0.64) | <0.001 | 0.78 (0.74–0.81) | <0.001 | 0.73 (0.70–0.76) | <0.001 | 0.81 (0.79–0.84) | <0.001 | 0.61 (0.57–0.66) | <0.001 | 0.83 (0.77–0.89) | <0.001 |
| 2018 | 1.03 (1.01–1.04) | 0.002 | 0.97 (0.95–0.98) | <0.001 | 1.01 (0.98–1.05) | 0.462 | 0.99 (0.96–1.01) | 0.339 | 0.97 (0.95–0.99) | 0.014 | 0.91 (0.87–0.94) | <0.001 | 0.96 (0.91–1.02) | 0.169 |
| 2023 | 1.02 (1.00–1.04) | 0.12 | 0.85 (0.84–0.87) | <0.001 | 0.98 (0.94–1.02) | 0.391 | 0.95 (0.92–0.98) | 0.003 | 1.03 (1.00–1.05) | 0.029 | 0.93 (0.89–0.97) | 0.001 | 0.95 (0.87–1.03) | 0.209 |
| *Insufficient fruit and vegetable intake* |  |  |  |  |  |  |  |  |  |  |  |  |  |  |
| 2007 (Reference) | 1 |  | 1 |  | 1 |  | 1 |  | 1 |  | 1 |  | 1 |  |
| 2018 | 0.98 (0.97–0.99) | 0.001 | 0.98 (0.97–0.99) | <0.001 | 0.99 (0.96–1.01) | 0.316 | 0.92 (0.90–0.94) | <0.001 | 0.93 (0.92–0.95) | <0.001 | 1.21 (1.14–1.27) | <0.001 | 1.04 (0.98–1.10) | 0.221 |
| 2023 | 1.09 (1.07–1.10) | <0.001 | 1.03 (1.02–1.05) | <0.001 | 1.14 (1.11–1.17) | <0.001 | 1.08 (1.05–1.10) | <0.001 | 0.97 (0.96–0.99) | 0.001 | 1.12 (1.05–1.19) | <0.001 | 1.20 (1.13–1.28) | <0.001 |

*Note.* aPR = adjusted prevalence ratio. Estimates derived from adjusted survey-weighted Poisson regression models with robust standard errors. Overall models were adjusted for sex, age group, and geographic region. Sex-stratified models were adjusted for age group and geographic region. The earliest available survey year for each behavioural outcome was the reference category.

**Supplementary Table S9**

*Sensitivity Analysis for Insufficient Physical Activity*

| **Variable** | **Primary analysis**  **aPR (95% CI)** | **Excluding 2013**  **aPR (95% CI)** |
| --- | --- | --- |
| 2007 (reference) | 1 | 1 |
| 2013 | 0.70 (0.68–0.71) | - |
| 2018 | 1.01 (1.00–1.02) | 1.01 (0.99–1.01) |
| 2023 | 0.95 (0.94–0.96) | 0.94 (0.93–0.95) |

| **Interaction test** | **Primary analysis**  ***p* value** | **Excluding 2013**  ***p* value** |
| --- | --- | --- |
| Survey year × sex | <0.001 | <0.001 |
| Survey year × age group | <0.001 | <0.001 |
| Survey year × region | <0.001 | <0.001 |

*Note*. aPR = adjusted prevalence ratio. Prevalence ratios were estimated using survey-weighted Poisson regression with robust variance and adjusted for sex, age group, and geographic region. Interaction p values were obtained from joint Wald tests. The sensitivity analysis excluded all observations from the 2013 survey.

**Supplementary Table S10.**

*Survey-Weighted Prevalence of Current Smoking and Chewing Tobacco Among Indonesian Adolescents, 2013–2023*

| **Characteristic** | **Smoking** | | | **Chewing** | | |
| --- | --- | --- | --- | --- | --- | --- |
|  | **2013**  **% (95% CI)** | **2018**  **% (95% CI)** | **2023**  **% (95% CI)** | **2013**  **% (95% CI)** | **2018**  **% (95% CI)** | **2023**  **% (95% CI)** |
| Overall | 7.25 (7.05–7.44) | 9.10 (8.87–9.32) | 7.42 (7.11–7.74) | 2.50 (2.38–2.62) | 1.14 (1.07–1.21) | 0.88 (0.80–0.95) |
| Male | 14.00 (13.63–14.37) | 17.45 (17.04–17.86) | 14.26 (13.69–14.84) | 2.46 (2.31–2.61) | 1.09 (0.99–1.18) | 0.90 (0.79–1.01) |
| Female | 0.18 (0.14–0.23) | 0.30 (0.24–0.36) | 0.16 (0.11–0.21) | 2.54 (2.39–2.70) | 1.19 (1.09–1.29) | 0.86 (0.77–0.95) |
| 10–12 years | 0.39 (0.32–0.47) | 0.58 (0.50–0.67) | 0.25 (0.16–0.34) | 2.31 (2.14–2.47) | 0.93 (0.83–1.03) | 0.79 (0.68–0.90) |
| 13–15 years | 5.07 (4.79–5.36) | 6.71 (6.37–7.04) | 5.09 (4.65–5.53) | 2.57 (2.39–2.74) | 1.17 (1.06–1.28) | 0.83 (0.71–0.95) |
| 16–18 years | 18.88 (18.34–19.42) | 20.61 (20.06–21.16) | 17.22 (16.44–18.00) | 2.68 (2.48–2.88) | 1.33 (1.19–1.47) | 1.02 (0.89–1.15) |
| Sumatera | 6.53 (6.23–6.83) | 7.74 (7.40–8.08) | 4.84 (4.48–5.20) | 2.56 (2.37–2.75) | 1.22 (1.10–1.35) | 0.79 (0.65–0.92) |
| Jawa | 7.97 (7.65–8.29) | 10.29 (9.91–10.66) | 9.40 (8.86–9.94) | 1.79 (1.63–1.94) | 0.59 (0.50–0.68) | 0.29 (0.22–0.37) |
| Kepulauan Nusa Tenggara | 6.02 (5.53–6.51) | 8.60 (8.00–9.20) | 6.93 (6.12–7.75) | 5.26 (4.63–5.89) | 3.49 (3.02–3.97) | 2.83 (2.37–3.28) |
| Kalimantan | 5.21 (4.78–5.64) | 6.88 (6.36–7.40) | 4.52 (3.92–5.12) | 2.23 (1.87–2.60) | 1.07 (0.87–1.28) | 0.63 (0.45–0.81) |
| Sulawesi | 7.37 (6.92–7.82) | 8.16 (7.69–8.62) | 5.75 (5.23–6.27) | 2.61 (2.33–2.90) | 0.65 (0.52–0.79) | 0.57 (0.43–0.71) |
| Kepulauan Maluku | 6.07 (5.14–7.00) | 6.91 (5.99–7.84) | 4.16 (3.23–5.09) | 5.68 (4.72–6.63) | 3.39 (2.81–3.97) | 1.52 (1.06–1.98) |
| Papua | 5.26 (4.36–6.17) | 6.44 (5.53–7.36) | 4.47 (3.40–5.54) | 12.05 (9.84–14.26) | 9.69 (8.15–11.24) | 13.60 (11.52–15.69) |

*Note.* Smoking and chewing tobacco were assessed separately from 2013 onward. In 2007, smoking and chewing tobacco were assessed using a combined item and therefore could not be disaggregated.

**Supplementary Table S11.**

*Smoking-Only Sensitivity Analysis, 2013–2023*

| **Survey year** | **aPR (95% CI)** |
| --- | --- |
| 2013 (reference) | 1 |
| 2018 | 1.15 (1.11–1.18) |
| 2023 | 0.92 (0.88–0.96) |

| **Interaction tests** | ***p* value** |
| --- | --- |
| Survey year × sex | 0.044 |
| Survey year × age group | <0.001 |
| Survey year × region | <0.001 |

*Note.* aPR = adjusted prevalence ratio. Estimates were obtained using survey-weighted Poisson regression with robust variance and adjusted for sex, age group, and geographic region. Interaction *p* values were obtained using joint Wald tests. The analysis was restricted to 2013–2023 because smoking could not be separated from chewing tobacco in the 2007 survey.

**Then Supplementary Table S12.**

*Sensitivity Analysis Comparing Adjusted Prevalence Ratios Using Original and Rescaled Survey Weights*

| **Year vs reference** | **Original**  **aPR (95% CI)** | **Rescaled**  **aPR (95% CI)** |
| --- | --- | --- |
| *Tobacco use* | | |
| 2007 (Reference) | 1 | 1 |
| 2013 | 1.17 (1.14–1.21) | 1.17 (1.14–1.21) |
| 2018 | 1.15 (1.12–1.19) | 1.15 (1.12–1.18) |
| 2023 | 0.93 (0.89–0.96) | 0.92 (0.89–0.96) |
| *Alcohol use* | | |
| 2007 (Reference) | 1 | 1 |
| 2018 | 0.95 (0.88–1.02) | 0.96 (0.89–1.03) |
| 2023 | 0.41 (0.37–0.46) | 0.42 (0.37–0.47) |
| *Insufficient physical activity* | | |
| 2007 (Reference) | 1 | 1 |
| 2013 | 0.70 (0.68–0.71) | 0.69 (0.68–0.70) |
| 2018 | 1.013 (1.004–1.022) | 1.007 (0.998–1.016) |
| 2023 | 0.946 (0.935–0.957) | 0.941 (0.930–0.952) |
| *Insufficient fruit and vegetable intake* | | |
| 2007 (Reference) | 1 | 1 |
| 2018 | 0.98 (0.97–0.98) | 0.977 (0.971–0.983) |
| 2023 | 1.05 (1.05–1.06) | 1.055 (1.048–1.062) |

**Supplementary Table S13.**

*Overall Survey-Weighted and Marginal Adjusted Prevalence of Behavioural Risk Factors, 2007 –2023*

| **Year** | **Survey-weighted**  **% (95% CI)** | **Marginal adjusted**  **% (95% CI)** |
| --- | --- | --- |
| *Tobacco use* | | |
| 2007 | 7.84 (7.67–8.00) | 8.56 (8.38–8.73) |
| 2013 | 9.49 (9.27–9.71) | 10.02 (9.81–10.24) |
| 2018 | 10.08 (9.84–10.31) | 9.87 (9.67–10.07) |
| 2023 | 8.19 (7.87–8.51) | 7.91 (7.64–8.18) |
| *Alcohol use* | | |
| 2007 | 1.77 (1.69–1.85) | 1.74 (1.65–1.83) |
| 2018 | 1.63 (1.54–1.72) | 1.65 (1.56–1.74) |
| 2023 | 0.72 (0.64–0.80) | 0.71 (0.63–0.79) |
| *Insufficient physical activity* | | |
| 2007 | 66.96 (66.55–67.37) | 65.52 (65.08–65.96) |
| 2013 | 45.90 (45.26–46.53) | 45.60 (44.97–46.23) |
| 2018 | 66.17 (65.72–66.61) | 66.38 (65.93–66.83) |
| 2023 | 61.78 (61.13–62.43) | 62.00 (61.36–62.65) |
| *Insufficient fruit and vegetable intake* | | |
| 2007 | 79.34 (78.96–79.72) | 80.10 (79.70–80.51) |
| 2018 | 78.27 (77.92–78.63) | 78.26 (77.91–78.62) |
| 2023 | 84.47 (84.07–84.88) | 84.48 (84.08–84.89) |

*Note.* Marginal adjusted prevalence estimates were derived from survey-weighted Poisson regression models adjusted for sex, age group, and geographic region. Alcohol use and insufficient fruit and vegetable intake were unavailable in the released 2013 dataset.
